# Agave-Derived Carbon Dots Incorporated into PVA/Chitosan Nanofibers for Prolonged Antibacterial Activity

**DOI:** 10.1021/acsomega.5c10072

**Published:** 2026-05-30

**Authors:** Mitzi Nayeli Sánchez-De la Cruz, Marilú Chávez-Castillo, Edna Vázquez-Vélez

**Affiliations:** † 87782 Lab. Biofísica, Instituto de Ciencias Físicas, Universidad Nacional Autónoma de México, Av. Universidad s/n, Col. Chamilpa, Cuernavaca, Morelos 62210, México; ‡ 227134 División Académica de Mecánica Industrial, Universidad Tecnológica Emiliano Zapata del Estado de Morelos, México, Av. Universidad Tecnológica 1, Palo Escrito, Emiliano Zapata, Morelos 62765, México

## Abstract

The rise of antimicrobial-resistant pathogens, including drug-resistant *Escherichia coli* (*E. coli*), underscores the urgency of developing alternative antibacterial strategies. In this study, natural carbon dots (CDs) were synthesized from agave using a simple carbonization method and incorporated into poly­(vinyl alcohol)-chitosan (PVA/QS) nanofibrous scaffolds via electrospinning. The CDs exhibited blue fluorescence, a bandgap of 4.14 eV, and nanoscale dimensions, as confirmed by transmission electron microscopy (TEM), Raman spectroscopy, and dynamic light scattering (DLS). Fourier transform infrared (FTIR) analysis revealed surface functional groups (−OH, −NH_2_, −COOH, and −C=O) derived from the natural precursor. Their incorporation into the polymer matrix enhanced intermolecular interactions, resulting in uniform, porous, and hydrophilic nanofibers (NF) with improved structural stability. Scanning electron microscopy (SEM) confirmed nanofiber morphology, and field-emission transmission electron microscopy (FETEM) imaging revealed that the CDs appeared enriched toward the fiber periphery, suggesting a shell-like distribution likely driven by charge-induced migration during electrospinning. Antibacterial assays against *E. coli* showed a time-dependent increase in inhibition zones over 4 days, exceeding the single-day inhibition observed with gentamicin, indicating prolonged antibacterial activity. This effect is likely associated with the surface chemistry of the CDs, their interaction with bacterial membranes, and potential reactive oxygen species (ROS)-related mechanisms. Overall, the PVA/QS/CD nanofibers represent a promising biomaterial for topical antimicrobial applications, offering localized and sustained antibacterial activity while reducing reliance on conventional antibiotics.

## Graphical abstract

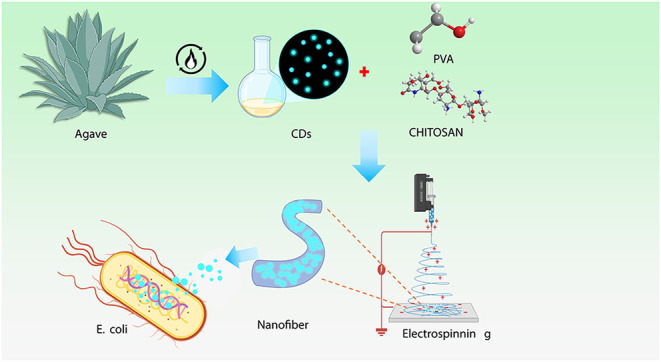

## 1 Introduction

The emergence of antimicrobial-resistant pathogens has become a significant global public health challenge.^1^ This issue has driven the search for innovative antibacterial technologies. Although antimicrobial resistance is not directly assessed in this study, it provides an essential context for the development of new materials with antibacterial potential.

*Escherichia coli* (*E. coli*) is increasingly exhibiting resistance to conventional antibiotics, a problem intensified by their widespread and sometimes inappropriate use. While *E. coli* is primarily a commensal organism inhabiting the gastrointestinal tract, it can also act as an extraintestinal pathogenic bacterium (ExPEC).^2^ Although cutaneous infections such as cellulitis or necrotizing soft tissue infections are relatively uncommon, they can be severe and often require hospitalization and prolonged antibiotic therapy.^3^ These challenges highlight the urgent need for alternative therapeutic strategies to manage infections and mitigate the progression of antibiotic resistance.

Current alternative strategies include bacteriophages, antimicrobial peptides, polymers, and carbon-based nanomaterials such as carbon dots (CDs).^4–6^ CDs can be synthesized from chemical precursors or natural sources and have attracted increasing attention for their antimicrobial and anticancer properties, low toxicity, water solubility, biocompatibility, and functional versatility.^7–10^ Their antimicrobial activity may involve multiple mechanisms, including physical disruption of membranes, interference with protein or enzyme activity, DNA damage, generation of reactive oxygen species (ROS), and synergistic effects under photothermal or photodynamic activation. These multifaceted mechanisms reduce the likelihood of bacterial resistance, making natural CDs promising candidates for next-generation antimicrobial agents.^10,11^ However, despite their potential, the biomedical use of natural CDs is often limited by aggregation and degradation, which compromise their stability, efficacy, and safety.^12,13^ To address these limitations, surface functionalization with hydrophilic groups or polymer chains has been proposed to improve the dispersibility of CDs and reduce interparticle interactions.^14,15^

In this context, nanofibers (NF) have emerged as excellent platforms for incorporating CDs because of their high surface-to-volume ratio, porosity, and mechanical strength. Their morphology also mimics that of the extracellular matrix (ECM), making them ideal scaffolds for the controlled release of the active molecules. Previous studies have explored the loading of CDs into nanofibers by using various polymeric matrices. For example, CDs loaded into poly­(*N*-isopropylacrylamide) (PNIPAAm)/poly­(methyl methacrylate) (PMMA) blend nanofibers enable thermally triggered release, providing stability, and can be released upon thermal stimulation.^16^ Nylon-11/CD nanofibers exhibit enhanced mechanical properties and cytocompatibility.^17^ Polyacrylonitrile (PAN)/CD scaffolds promote reepithelialization and provide antimicrobial protection during tissue regeneration,^18^ and natural CDs from pomegranate peel, incorporated into poly­(vinyl alcohol) (PVA)/oxidized alginate nanofibers, improve fibroblast proliferation and protect the matrix against oxidation.^19^ Similarly, PAN/CD nanofibers have shown broad-spectrum antibacterial activity via photodynamic inactivation.^20^

The above findings underscore the need to develop nanofibrous systems based on biodegradable, biocompatible polymers, such as poly­(vinyl alcohol) and chitosan, combined with natural CDs derived from renewable natural sources for wound-dressing applications. Although agave CDs have been previously reported via pyrolysis-based synthesis, no study has reported the production of agave-derived CDs via carbonization or their incorporation into biodegradable PVA/QS nanofibrous scaffolds for antimicrobial applications. The release of natural CDs from nanofibers prolonged antibacterial activity against *E. coli* for up to 3 days, as demonstrated by the expanding inhibition zones, highlighting their potential for localized, sustained antimicrobial action. This approach represents a promising alternative for preventing skin infections, reducing the risk of resistance development, and minimizing the need for repeated application of antibiotics.

## 2 Experimental Section

### 2.1 Materials

The reagents used in this study were analytical grade poly­(vinyl alcohol) (PVA) [−CH_2_CHOH−]*_n_*, MW 89,000–98,000, and chitosan (QS) of medium molecular weight from Sigma-Aldrich. The solvents used were acetic acid (CH_3_COOH) [MW = 60.05 g/mol] from Sigma-Aldrich and deionized water from Meyer. The reagent for contact angle (CA) measurement was diiodomethane (CH_2_I_2_) [MW = 267.84 g/mol] from Sigma-Aldrich.

### 2.2 Green Synthesis of CDs

A blue agave leaf (*Agave Tequilana Weber*) was used to synthesize carbon quantum dots. The process began with cleaning the leaf and removing the outer skin to obtain only the fiber. Once the fiber was cut, 5 g was placed in porcelain crucibles for carbonization in a muffle furnace at 300 °C for 4 h.

The resulting carbon was ground in an agate mortar, transferred to an Eppendorf tube, mixed with 30 mL of deionized water, and ultrasonicated for 15 min at 40 kHz. Subsequently, the solution was filtered using medium-pore filter paper and then through filters with pore sizes of 400 nm and, finally, 200 nm (see Figure 1).

**Figure 1:**
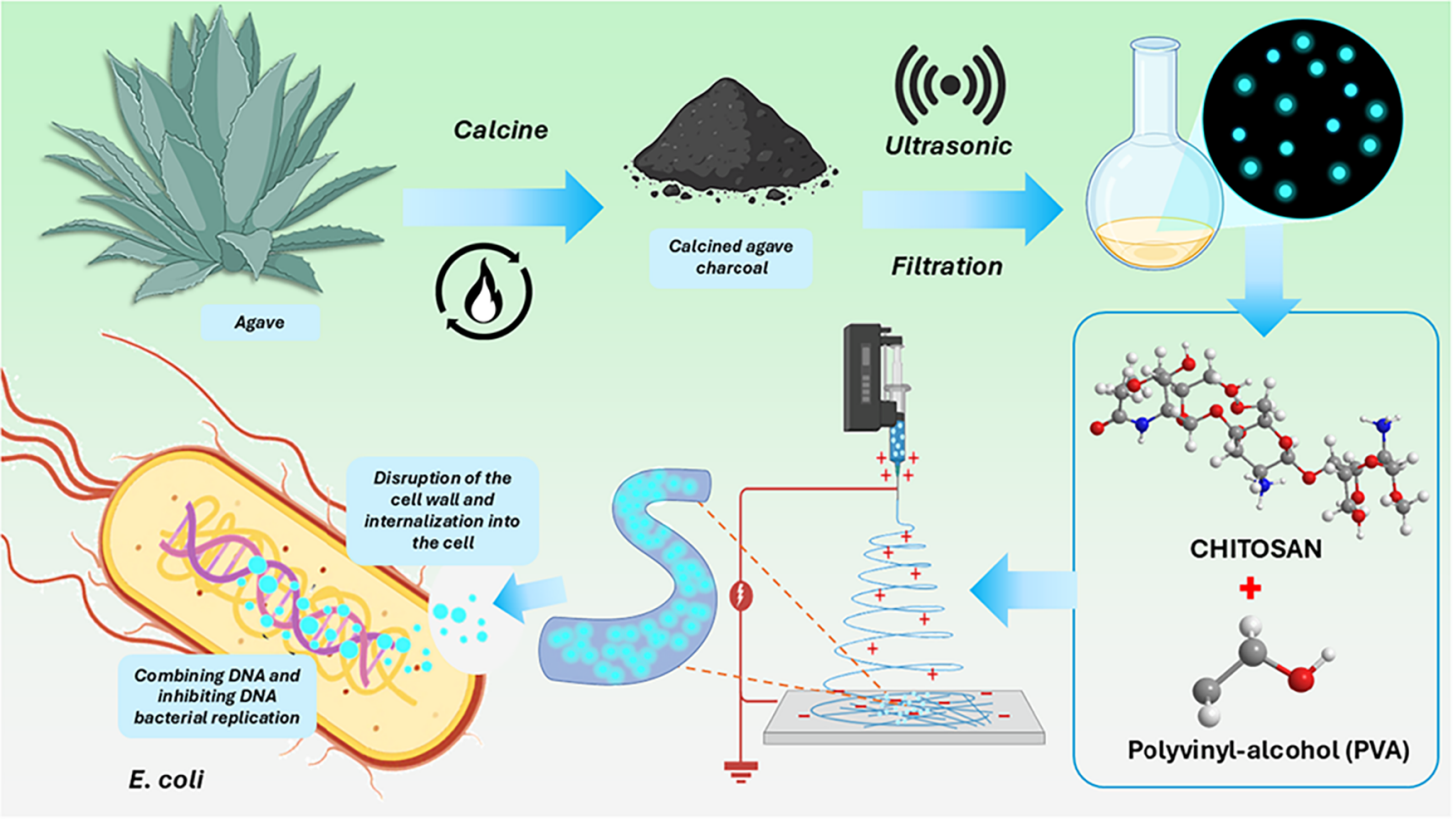
Green synthesis of natural CDs and their incorporation into PVA/QS nanofibers for antibacterial release.

### 2.3 Preparation of PVA/QS/CDs Nanofibers

To obtain the nanofibers, 10 mL of a 12% (w/v) poly­(vinyl alcohol) (PVA) solution was prepared and stirred magnetically at 40 °C until fully dissolved. Separately, 10 mL of a 2% (w/v) chitosan (QS) solution in acetic acid–water (9:1 v/v) was prepared under constant stirring until it was fully dissolved. Solutions were aged for 3 days before electrospinning to facilitate alterations in chain conformation and intermolecular interactions (e.g., hydrogen bonding and partial gelation in chitosan), thereby enhancing effective viscosity and affecting charge distribution. These time-dependent modifications were evidenced by an increase in conductivity from 752 to 773 μS·cm^–1^ in the PVA solution, a factor known to augment electrohydrodynamic stability and fiber formation during electrospinning.^21^

Then, the electrospinning solution was formulated by blending 5 mL of the PVA solution (12% w/v), 1 mL of the QS solution (2% w/v), and 1 mL of the CDs solution [125 mg/mL], thereby establishing a PVA/QS/CDs ratio of 1:0.03:0.2. The resulting mixture was loaded into a syringe equipped with a 21G needle and electrospun at a flow rate of 0.45 mL/h. Electrospinning was performed at an applied voltage of 27–29 kV, 27 °C, and a needle-to-collector distance of 14 cm. The collector was kept stationary.

### 2.4 Characterization of CDs

The optical properties of the CDs were evaluated using fluorescence spectroscopy with a luminescence spectrophotometer (Aminco Bowman Series 2 model). Emission spectra were recorded at various excitation wavelengths to determine the emission-maximum range. UV–vis spectroscopy was used to determine the maximum absorption wavelength of the natural CDs with an Ocean Optics spectrophotometer (SR-6 model, Orlando, FL, USA). The optical bandgap (*E*_g_) of the CDs was determined from their absorbance data using the Tauc plot method.^22^ The absorption coefficient (α) was calculated by eq 1

1 $$\alpha =\frac{2.303\cdot a}{d}$$

where *a* is the absorbance and *d* is the cuvette thickness (cm). The relationship between photon energy (*h*ν) and the absorption coefficient is expressed by eq 2.

2 $${\left(\alpha h\nu \right)}^{1/n}=A\left(h\nu -E_{g}\right)$$

where *A* is the electronic transition constant and *n* depends on the type of transition (*n* = 1/2 for direct, *n* = 2 for indirect). Wavelength values were converted to photon energies and ${\left(\alpha h\nu \right)}^{1/n}$ plotted against *h*ν. The linear portion of the plot was extrapolated to (α*h*ν)^1/*n*^ = 0, giving the *E* _g_ value.

The chemical composition and surface functional groups of the agave-derived CDs were analyzed using attenuated total reflectance (ATR) and Fourier transform infrared (FTIR) spectroscopy on a Bruker α II. Spectra were collected in the 400–4000 cm^–1^ range, with a resolution of 4 cm^–1^ and 16 scans. Raman spectroscopy was employed to investigate the graphitic carbon structure of the CDs using a confocal Raman spectrometer (Bruker Senterra II, Ettlingen, Germany). The analysis was performed using the Surface Enhanced Raman scattering (SERS) technique, as previously reported.^23^ Silver nanoparticles (AgNPs) were deposited on silicon wafers to improve the plasmonic signal of the CDs. Raman measurements were conducted using a 785 nm laser at 25 mW and an integration time of 20,000 ms.

Morphological and size characterization of the CDs was performed by transmission electron microscopy (TEM) using a Zeiss microscope (Oberkochen, Germany) operated at 100 kV. For this, a CD solution (10 μL) was placed on a carbon-coated copper grid, followed by a drop of uranyl acetate (2%). Dynamic light scattering (DLS) was used to analyze the hydrodynamic size of CDs and their stability, as measured by zeta potential (ZP), using a Zetasizer Nano ZS-90 (Malvern, Worcestershire, U.K.).

### 2.5 Surface Free Energy of the Nanofibers

The surface free energy of the nanofibers (NF) was determined using the Owens-Wendt geometric mean method. For each probe liquid *l* (with known total surface tension γ*_l_* and dispersive and polar components γ*_l_* ^d^ and γ*_l_* ^p^), the relationship used is given in eq 3.^24^ Defining eq 3 by dividing $\sqrt{\gamma_{l}^{d}}$ , as a linear system (*y* = *mx* + *b*), eq 4.^25,26^ The surface free energy (γ_s_) is calculated from the contact angles measured for water and diiodomethane and surface tensions of standard liquids (Table 1). By solving the linear system to obtain *x* and *y*, then γ_s_ = γ_s_^d^ + γ_s_^p^.

3 $$\gamma_{l}\cdot \left(1+\cos\theta \right)=2\sqrt{\gamma_{s}^{d}\gamma_{l}^{d}}+\sqrt{\gamma_{s}^{p}\gamma_{l}^{p}}$$

4 $$\frac{\gamma_{l}\cdot \left(1+\cos\theta \right)}{2\sqrt{\gamma_{l}^{d}}}=\sqrt{\gamma_{s}^{d}}+\sqrt{\gamma_{s}^{p}\gamma_{l}^{p}}\sqrt{\frac{\gamma_{l}^{p}}{\gamma_{l}^{d}}}$$

**Table 1:** Polar γ*_l_* ^p^ and Dispersive γ*_l_* ^d^ Components of the Surface Tensions of the Liquids γ*_l_* ^24^

liquids	γ*_l_* ^p^ (dyn/cm)	γ*_l_* ^d^ (dyn/cm)	γ*_l_* (dyn/cm)
distilled water	51.0	21.8	72.8
diiodomethane	0	50.8	50.8

The NF contact angle was measured using the sessile drop method.^24^ Drop images were captured with a MicroView 1000 digital microscope (model Px-537), and contact angle (CA) values were determined by geometric analysis using ImageJ software. Six measurements were taken for each NF sample, and the average value was reported. The standard error was calculated on the basis of the standard deviation.

### 2.6 Characterization of PVA/Qs/CDs Nanofibers

The chemical composition of the PVA/QS/CDs NF was analyzed using Raman spectroscopy with a SENTERRA II Raman microscope (Bruker Corporation, Ettlingen, Germany). A 785 nm laser with a power of 100 mW and an acquisition time of 10,000 ms was used to acquire spectra over the range 400–4000 cm^–1^. All spectra were baseline-corrected and normalized for further analysis.

The morphology and size of the PVA/QS NFs with and without CDs were evaluated using scanning electron microscopy (SEM), TESCAN VEGA from Kohoutovice, Czech Republic, operated at 5 kV. Before imaging, samples were coated with a thin carbon layer by using a sputter-evaporation coater. Fiber diameters correspond to the external diameter of individual nanofibers measured directly from SEM images using ImageJ software. A total of 100 randomly selected fibers from different regions were analyzed from Figure 8a,b. The histograms represent the statistical distribution of these measured diameters (in nm), and the mean value was obtained by a Gaussian fit. The homogeneity and distribution of CDs within the nanofibers were analyzed by field-emission transmission electron microscopy (FETEM) using a JEOL FAS TEM instrument (Akishima, Tokyo, Japan) operated at 200 kV.

### 2.7 Bacterial Inhibition of PVA/QS/CDs Nanofibers

Bacterial inhibition was evaluated using the agar diffusion assay. Luria-Miller’s LB agar medium was prepared and poured into Petri dishes, which were then inoculated with a Gram-negative bacterium, enterotoxigenic *Escherichia coli* (ETEC, strain HD092A), at a concentration of 3 × 10^8^ CFU/mL, adjusted according to the McFarland standard. A gentamicin disc (10 μg) was used as a positive control in one Petri dish, while PVA/QS nanofibers (blank control) and PVA/QS/CDs NF were placed in separate dishes. The plates were incubated at 37 °C for 24 h, after which the diameter of the inhibition zone was measured. They were further incubated under ambient light for an additional 3 days to evaluate prolonged bactericidal activity. However, by the fourth day, the agar had begun to dry out.

## 3 Results and Discussion

### 3.1 Optical Properties of CDs

The synthesis of carbon dots via carbonization is straightforward and requires minimal equipment, making it applicable to a wide range of materials. Therefore, it is a valuable approach and preferred choice for producing carbon dots with tailored properties.^7^ CDs from agave bagasse have been synthesized by pyrolysis up to 500 °C (2 h).^27^ In this work, we synthesized CDs from an agave plant by carbonization at 300 °C and analyzed their optical properties. It is widely accepted that the fluorescence properties of CDs are influenced by their size, surface defects, functional groups, and oxidation state. CDs undergo charge separation, generating electron–hole pairs that become trapped at surface irregularities. The resulting excited states relax via fluorescence, a process that can also promote the formation of reactive oxygen species (ROS).^10^

The optical properties of the CDs were analyzed by using fluorescence spectroscopy. Emission spectra were collected at two excitation wavelengths to identify the maximum-emission range. The highest fluorescence intensities were observed under excitation at 345 and 381 nm (Figure 2a). When excited at 345 nm, the CDs showed a maximum emission at 431 nm, whereas excitation at 381 nm resulted in a shifted emission peak at 463 nm. In aqueous suspension, the CDs displayed an intense bluish fluorescence under UV illumination (381 nm), consistent with previous reports of blue-emitting CDs.^27–29^

**Figure 2:**
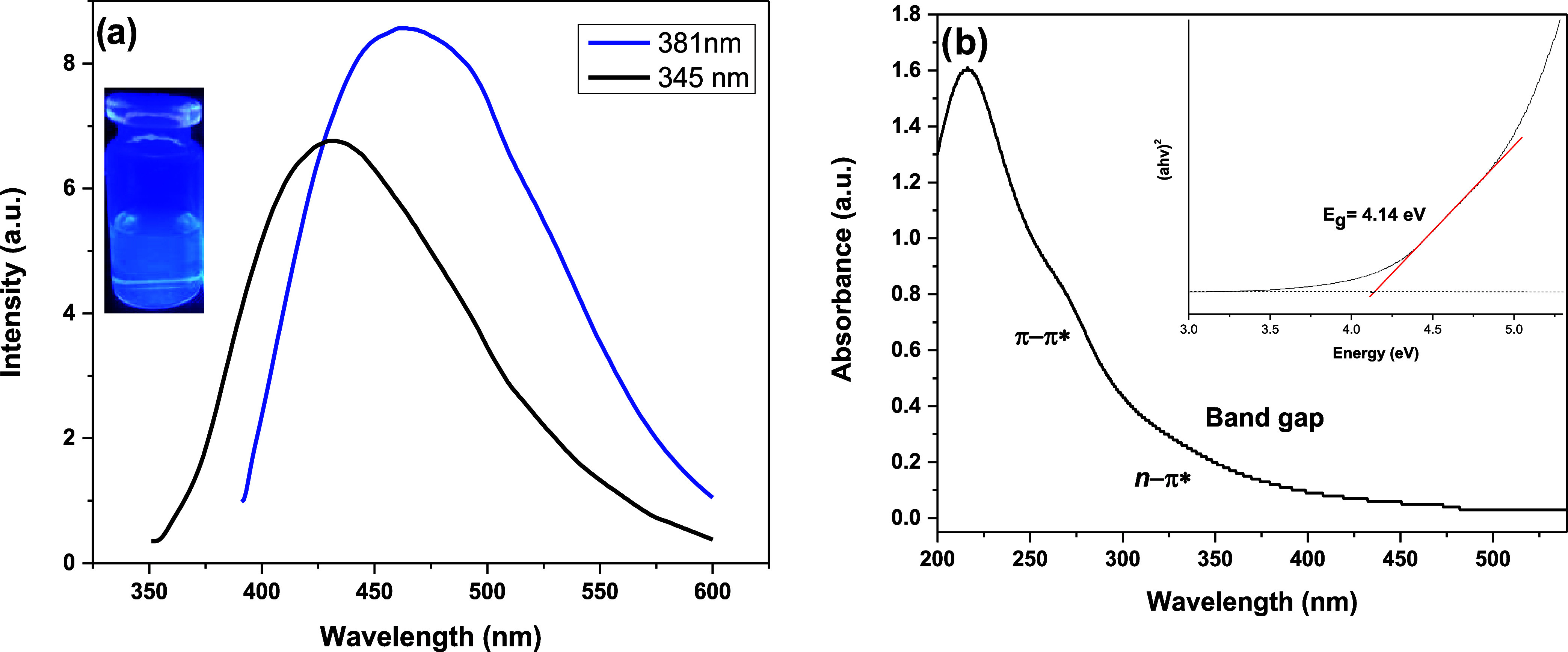
(a) Fluorescence and (b) UV–vis spectrum of CDs with the bandgap plot. Inset (a): Photograph of CDs solution from agave under irradiation UV light (380 nm).

The UV–vis absorption spectrum of CDs at 0.17 mg/mL revealed a prominent shoulder peak at 267 nm, corresponding to the sp^2^ hybridized π–π* transitions of C=C bonds in the aromatic core (see Figure 2b). A wide band at 329 nm was attributed to n−π* transitions of surface C=O groups.^27^ The optical bandgap (*E*_g_) was estimated using the Tauc plot method (eq 2).^20^ A linear fit with *r* = 2, indicative of a direct bandgap material, is shown in Figure 2b and is in agreement with previous studies on blue-emitting CDs.^30–32^ The *E*_g_ value was determined to be 4.14 eV from the *x*-intercept of the extrapolated linear region (red line). This relatively large bandgap suggests a very small particle size, consistent with quantum confinement effects of electron–hole pairs.

### 3.2 Physicochemical Characterization of CDs

TEM was used to examine the morphology and structure of the synthesized CDs. Figure 3 shows representative TEM images of the CDs, which appear to be spherical particles. In the micrograph with a 50 nm scale bar (Figure 3a), both cluster and scattered CDs are visible, and their histogram (Figure 3b) indicates an average diameter of 4.47 nm. At higher resolution (20 nm scale, Figure 3c), the CDs tend to align in fibrillar structures. At further magnification (10 nm scale), individual CDs with sizes near 1 nm can be distinguished. These structural features are consistent with the previously determined optical bandgap, as the small particle size supports quantum confinement effects. Similar morphologies and size ranges have been reported for CDs synthesized from natural sources through carbonization.^31,33^

**Figure 3:**
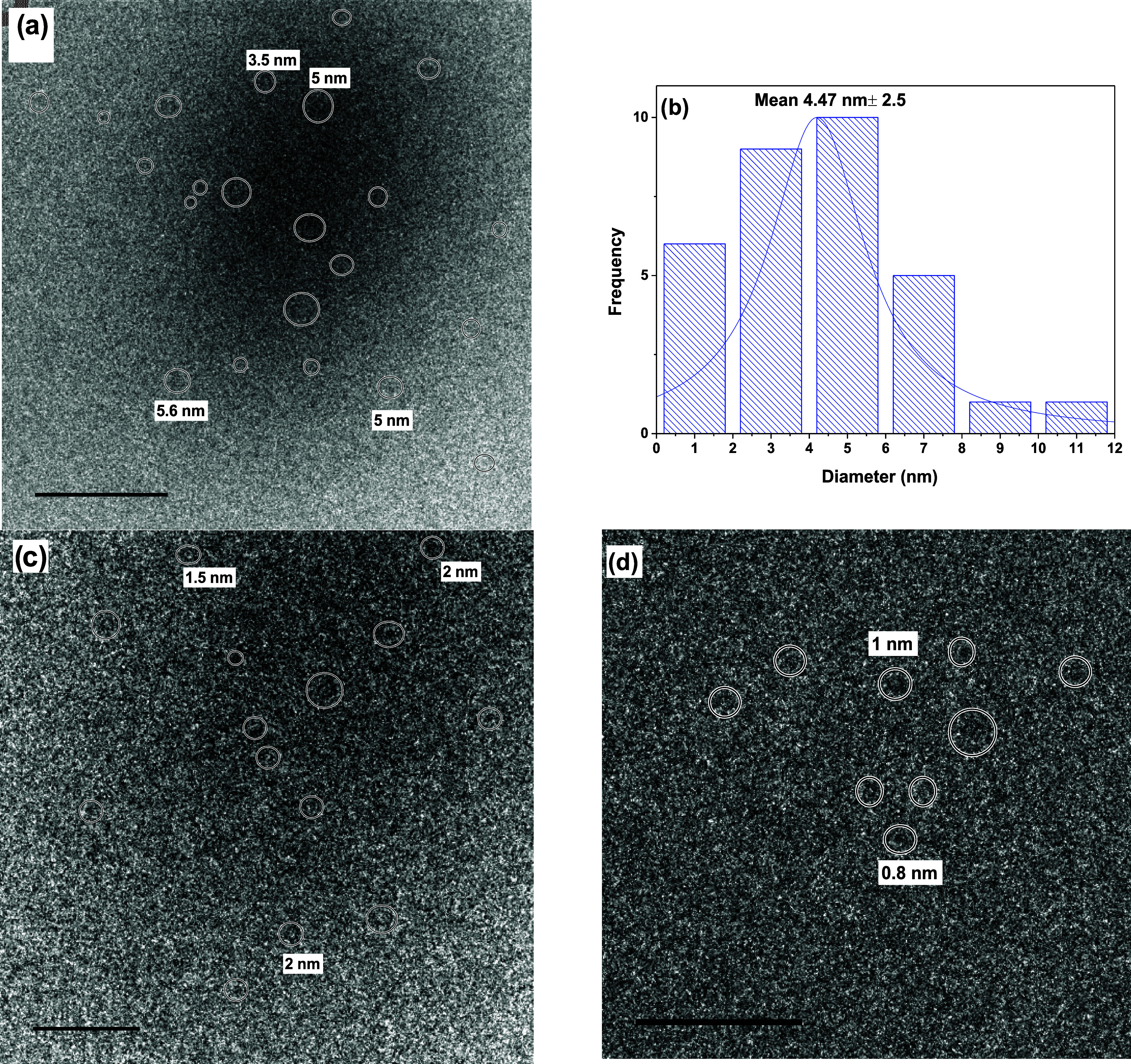
TEM micrographs of agave-derived CDs: (a, c) at 100 kX; (b) particle size distribution corresponding to micrograph (a); and (d) at 300 kX.

The Raman spectrum (Figure 4a) analyzed by SERS shows a prominent G band at 1572 cm^–1^, characteristic of the E2g vibrational mode of graphite, arising from the sp^2^-hybridized carbon atoms in a two-dimensional hexagonal lattice.^34^ A less intense D band is observed at 1326 cm^–1^, which is attributed to the vibrations of sp^3^-hybridized carbon atoms, reflecting the degree of defects and surface functionalization.^28^ The broad nature of these peaks is consistent with the TEM results, as such broadening is a typical feature of nanosized carbon particles.^35^

**Figure 4:**
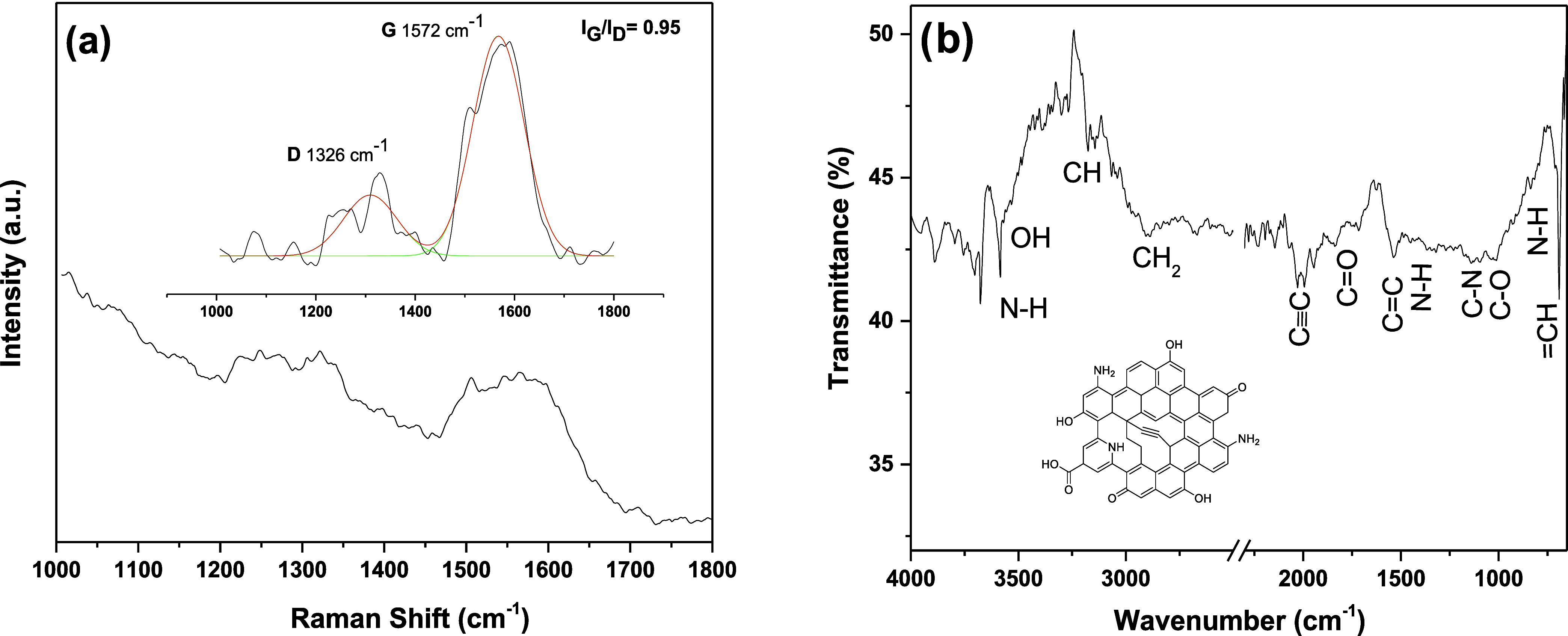
(a) Raman and (b) FTIR spectra of agave-derived CDs.

The G and D band intensities were obtained from the maximum peak counts after deconvolution of the Raman spectra. The relative intensity ratio (*I*_G_/*I*_D_) was approximately 0.95, reflecting the degree of graphitization. This value suggests that the CDs possess a graphitic crystalline structure, in agreement with previous reports on CDs synthesized from natural carbon sources.^31,35,36^ Furthermore, the ratio indicates a higher structural purity of the prepared CDs than in other studies that synthesized CDs by carbonization.^28^

The functional and biological properties of CDs are closely related to their core structure, particularly the functional groups exposed to the surface. To identify the functional groups originating naturally from the agave precursor and from the carbonization process, FTIR analysis was performed. As illustrated in Figure 4b, three characteristic N–H vibration bands were identified within the amine groups: a symmetric stretching vibration at 3580 cm^–1^, a strain band at 1537 cm^–1^, and a ripple vibration at 715 cm^–1^. A secondary N–H stretching band appears at 3677 cm^–1^, likely associated with a defect within a heterocyclic ring. A broad O–H stretching band was detected near 3500 cm^–1^, masked by the N–H signal. A faint band at approximately 3100 cm^–1^ corresponds to sp^2^ C–H stretching. While in the region close to 2900 cm^–1^, symmetric and asymmetric stretching vibrations of methylene groups, related to sp^3^-hybridized carbon, were observed. An unusual doublet in 2005 and 1995 cm^–1^ was assigned to a C≡C stretching vibration, likely derived from short sp-hybridized carbon chains embedded as defects between graphene-like aromatic domains. Two weak bands at 1880 and 1720 cm^–1^ were attributed to C=O stretching vibrations; the higher-frequency band could correspond to a carbonyl group in a warped environment or one with high conjugation restriction. A band at 1567 cm^–1^ was assigned to C=C stretching from the graphene-like domains. Other bands include a C–N stretching vibration at 1150 cm^–1^, a C–O stretching vibration at 1030 cm^–1^, and a =CH vibration at 689 cm^–1^.

These findings indicate that the CDs contain oxygenated functional groups, such as hydroxyl (O–H), C–O, and carbonyl C=O, as well as amines (NH_2_) and amides (CONH_2_). The surface chemistry of CDs depends strongly on the precursor composition and the synthetic method used. Agave tequilana is primarily composed of cellulose (mainly glucans), hemicellulose (mainly xylans), and lignin, which are rich in carbon and oxygen atoms. In addition, bioactive compounds such as flavonoids (e.g., kaempferol and quercetin) are present in the leaves, while pyranones and pyrazines, which contain abundant nitrogen atoms, have been identified in the bagasse.^37^ Consequently, CDs derived from agave are expected to be functionalized mainly with amine, hydroxyl, and carbonyl groups. Similar surface functionalization has been reported for CDs obtained from agave bagasse via pyrolysis.^27^

DLS and ζ-potential were used to determine the hydrodynamic size and stability of the CDs. The CDs exhibited an average size of 4.4 nm ± 5 (Figure 5a) and an average ZP of −13.1 mV ± 3 (Figure 5b). The negative potential is consistent with their surface chemistry, as indicated by the presence of deprotonated oxygen groups (e.g., −COO^–^, −O^–^). The particle size for the PVA/QS mixture is 14 nm ± 2 (Figure 5c). It exhibits a very low positive ZP (Figure 5d) at 6.9 mV ± 5 and another at 48.07 mV ± 5 (9.2%) reflecting the low chitosan content in the mix (PVA: CS = 30:1). However, upon incorporation of the carbon dots into the PVA/QS mixture, the average size was 12.6 nm ± 4 (Figure 5e), and their ZP increases to positive values of 35.2 mV ± 5 and another of 67.8 mV ± 5 (Figure 5f), probably the second corresponds to PVA/QS agglomerates. This shift indicates that the QS component was protonated under the acidic conditions of the suspension (pH = 2.96 ± 0.02), forming −NH_3_^+^ groups. The interaction between the negatively charged CDs and the protonated chitosan likely caused electrostatic adsorption of chitosan onto the CD surface, effectively coating the nanoparticles and reversing their surface charge.^38^ Similar behavior has been described in polymer–nanoparticle systems, where even small amounts of QS significantly increase surface charge and colloidal stability due to its strong cationic character in acidic media.^39^

**Figure 5:**
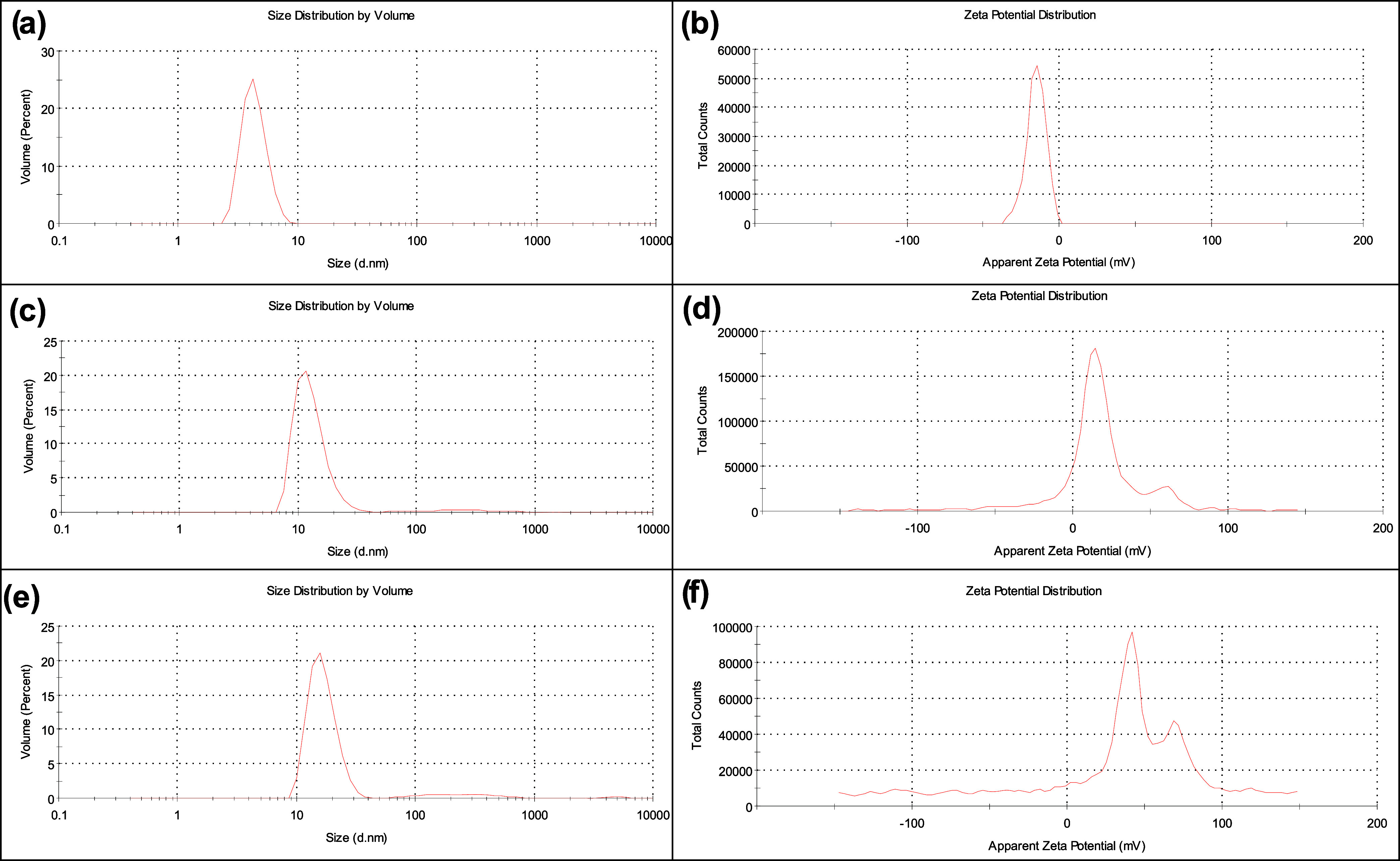
DLS and ZP of (a, b) agave-derived CDs solution, (c, d) PVA/QS solution, (e, f) PVA/QS/CDs solution, respectively.

In general, these results indicate that the ζ-potential of the system is highly sensitive to chitosan’s protonation state and its interactions with the carbon dots. Even at very low concentrations, QS dominates the surface charge under acidic conditions and induces strong positive stabilization of hybrid dispersion. This behavior is expected to influence not only colloidal stability but also electrohydrodynamic behavior during electrospinning.^40^

### 3.3 Surface Free Energy of Nanofibers

The surface free energy of the NFs was calculated using the Owens-Wendt method, including their polar and dispersive components, to relate their hydrophilicity to cell and protein adhesion on the polymer surface (biocompatibility potential).^41^ Contact angle (CA) measurements were performed to determine surface wettability. The PVA/QS NF exhibited a CA of 26.2°, indicating a highly hydrophilic surface (Figure 6a). Upon incorporation of CDs, the nanofibers exhibited a higher CA of 32.1° (Figure 6b), indicating decreased hydrophilicity due to the presence of CDs.

**Figure 6:**
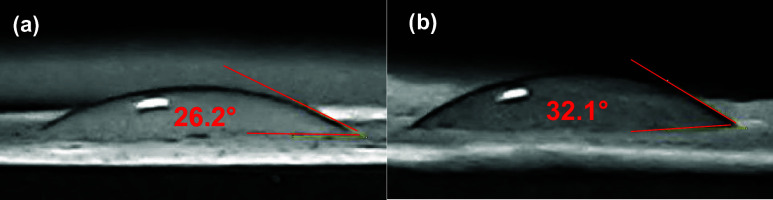
Contact angle of (a) PVA/QS and (b) PVA/QS/CDs nanofibers.

**Table 2:** Surface Free Energy with Its Polar and Dispersive Contributions for PVA/Qs and PVA/QS/CDs Nanofibers

nanofiber	SFE (mJ m^–2^)	ϒ polar (mJ m^–2^)	ϒ dispersive (mJ m^–2^)
PVA-Qs	72.16 ± 0.6	24.31 ± 1	47.85 ± 0.8
PVA-Qs-NCDs	68.97 ± 0.1	22.39 ± 0.7	46.58 ± 0.6

The PVA/QS/CDs NF exhibited a lower surface free energy compared to the fiber without CDs, with the principal reduction observed in the polar component (3.19 mJ m^–2^) (Table 2). Although the polar functional groups of the CDs (−OH, −NH_2_) were expected to increase the polar contribution, strong hydrogen-bond interactions with the polymer chains likely led to partial embedding of the CDs within the matrix. This interaction promotes a rearrangement in which the less polar carbonaceous domains preferentially orient toward the fiber surface, thereby reducing the polar component.^22^ The dispersive component decreased by 1.05 mJ m^–2^, suggesting morphological effects, such as surface pores or roughness, that hinder complete wetting.^42^ Despite these reductions, the PVA/QS/CDs NFs retained their hydrophilic character, a property essential for cellular biocompatibility in biomedical applications such as wound dressings.

### 3.4 Raman Analysis of Nanofibers

In the Raman spectrum of the PVA/QS NF shown in Figure 6a (black), a vibrational band corresponding to the O–H stretching mode was observed at 3125 cm^–1^. The symmetric and asymmetric C–H stretching vibrations from methylene groups and the polymer backbone appeared around 2900 cm^–1^. A methylene bending vibration was detected at 1450 cm^–1^, followed by a C–H bending mode at 1300 cm^–1^. The C–O stretching vibration was observed at 1100 cm^–1^, while the bands at 800 and 900 cm^–1^ were assigned to C–C and C–C–O vibrations, respectively. These spectral features confirm the chemical structure of the PVA polymer matrix forming the nanofiber. However, the vibrational bands of QS are not observable, likely due to its low concentration and the localized nature of Raman analysis.

In the same Raman spectrum of the PVA/QS/CDs NF (red), characteristic vibrational signals from both PVA and QDs are observed. A weak C–N stretching band appears near 1500 cm^–1^, accompanied by a C=C vibrational band at 1600 cm^–1^. Compared with pure PVA, the C–C vibration shifts to a lower wavenumber (790.6 cm^–1^), while the C–C–O band appears at 881 cm^–1^. These shifts may be associated with intermolecular hydrogen bonding between the functional groups of the CDs (−OH, −COOH, −NH_2_, etc.) and the PVA/QS chains, which suggests possible changes in the local electronic environment (see the molecular structure spectra in refs 43,44 ).

Additionally, new bands in the 1700–2100 cm^–1^ region may be attributed to combination or second-order modes, as well as overtones associated with graphitic sp^2^ domains in the CDs.^45^ Interestingly, the symmetric and asymmetric stretching bands of the methylene (C–H) groups, typically observed around 2900 cm^–1^, are no longer visible. This disappearance may be associated with intermolecular hydrogen bonding between the hydroxyl groups of PVA and the oxygen- or nitrogen-containing functional groups present on the surface of the CDs, as illustrated in the molecular structure image from the Raman spectrum.^46,47^ Such interactions perturb and attenuate the vibrational modes of CH_2_, leading to their absence in the Raman spectra. The emergence of additional C–O bands around 1100 cm^–1^ provides further support for these intermolecular interactions.

### 3.5 Microscopy of Nanofibers

SEM analysis was performed to evaluate the morphology and size of the nanofibers. Figure 7 presents the SEM images of (a) PVA/QS/NF and (b) PVA/QS/CDs NF. Both systems exhibit a scaffold-like morphology with average diameters of 378 and 388 nm, respectively. The incorporation of CDs results in slightly larger fibers with flatter strand features. Histograms confirm a narrow size distribution, indicating a good uniformity in both samples. This scaffold-like architecture is particularly relevant for wound-dressing applications, as it closely resembles the structure and function of the extracellular matrix (ECM).^48,49^ Furthermore, interactions between polymer chains, as indicated by Raman spectroscopy, together with increased fiber planarity, are expected to contribute to the prolonged antibacterial activity of embedded CDs.

**Figure 7:**
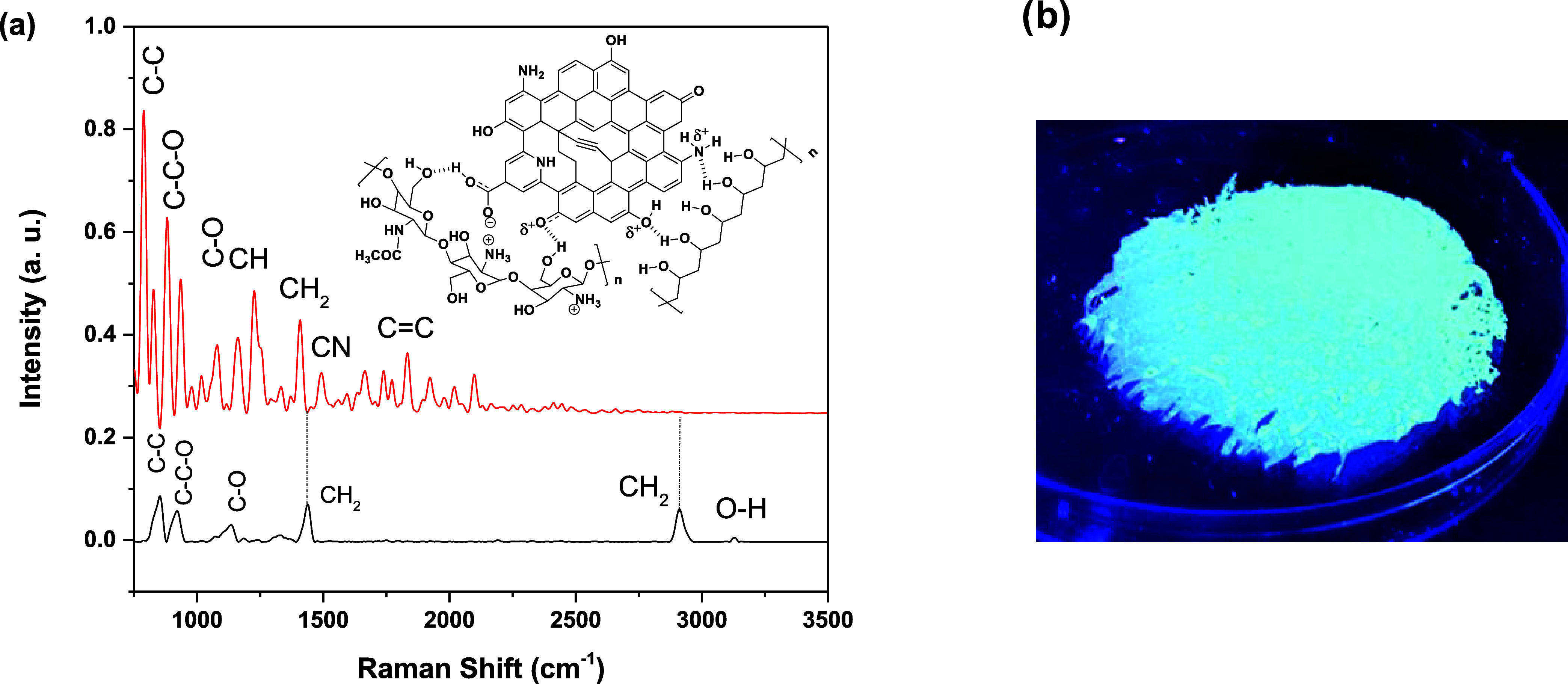
(a) Raman spectrum and (b) image under UV light (λ = 380 nm) of PVA/QS/CDs NF.

To elucidate the distribution of CDs within the PVA/QS NF, FETEM analysis was carried out. Figure 8 (above) presents micrographs at a 50 nm scale, illustrating the nanofiber diameter. Interestingly, in both TEM micrographs, a darker contrast is observed toward the periphery of the nanofibers compared with the core. Although the origin of this radial contrast distribution cannot be conclusively determined from the present data, it may be associated with differences in the local composition or density within the fibers. ζ-Potential measurements indicate a strong positive surface charge in the PVA/QS/CD system under acidic conditions, suggesting electrostatic interactions between negatively charged CDs and protonated chitosan chains. Under electrospinning conditions, charge distribution and electrohydrodynamic forces may influence nanoparticle redistribution within the jet.^50^

**Figure 8:**
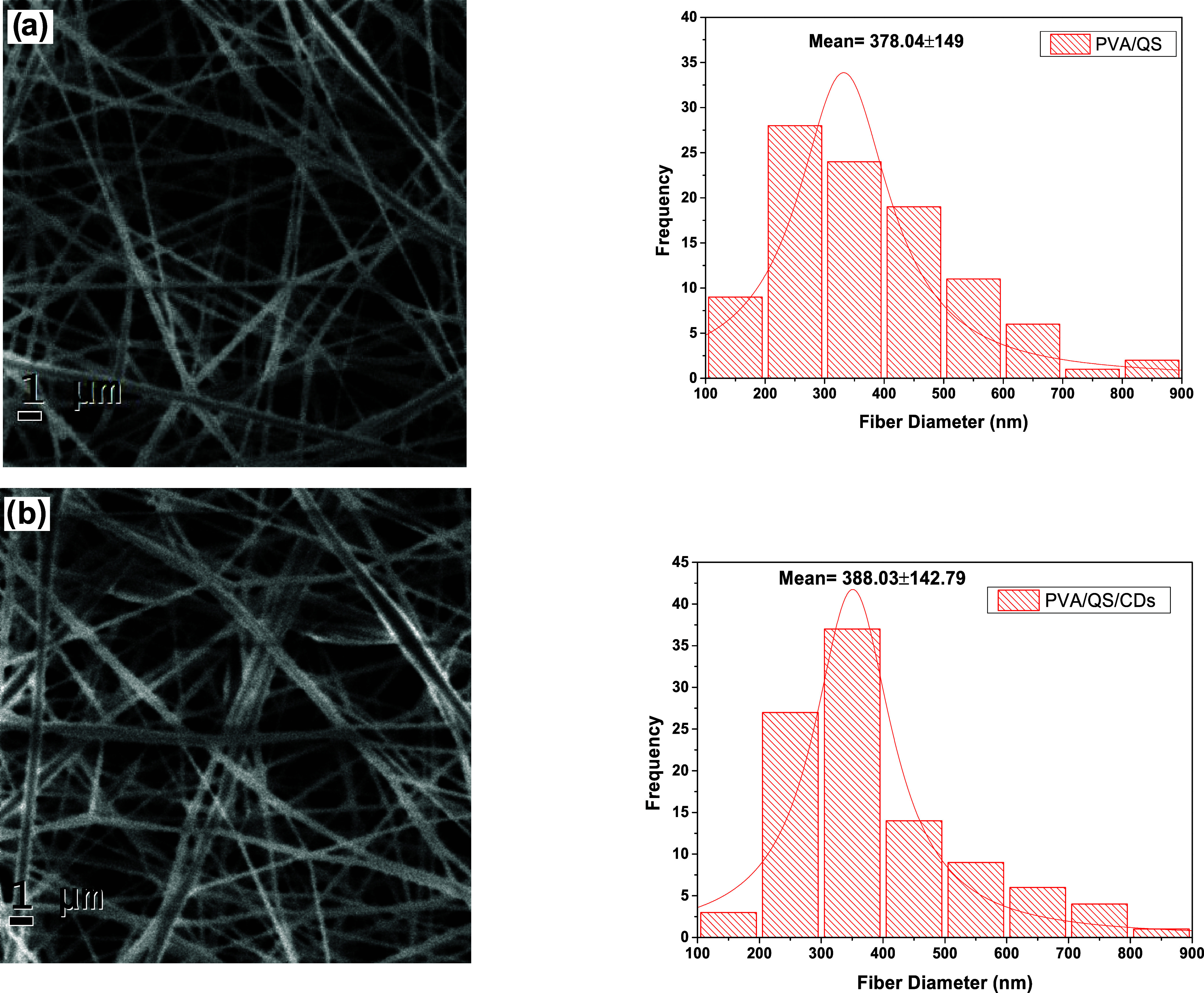
SEM Image of (a) PVA/QS NF and (b) PVA/QS/CDs NF (left) and their histograms, respectively (right).

From a micromechanical perspective, a stiffer outer layer functions as reinforcement for the cladding, thereby increasing the fiber’s effective modulus and influencing its failure mode. Research indicates that this effect is predominantly governed by the relative layer thickness and the phase modulus contrast, and it becomes increasingly significant as the fiber diameter diminishes.^51,52^

On the other hand, this peripheral enrichment of CDs likely contributes to their release behavior and antibacterial performance as CDs near the surface are more accessible to the medium and bacterial cells. This also facilitates a faster initial diffusion of the CDs while maintaining a sustained release from the internal polymer matrix.

At higher magnification (20 nm scale), the nanofiber surface appears rough, with several CDs approximately 6 nm in diameter aligned along the fiber. Further magnification (on the 10 nm scale) reveals additional CDs with dimensions of ∼2 nm.^53^

### 3.6 Bacterial Inhibition Analysis of PVA/QS/CDs Nanofibers

Bacterial inhibition assays against *E. coli* were performed using PVA/QS/CDs NFs, both with and without CDs, and compared with the antibiotic gentamicin. As shown in Figure 9a, the PVA/QS NF did not produce an inhibition halo. Although chitosan is known for its antimicrobial properties,^54,55^ the low QS concentration (0.03%) in the composite appears insufficient to exert significant antimicrobial activity. Research on the antimicrobial activity of chitosan indicates that higher concentrations lead to greater bacterial inhibition.^56^ Upon incorporation of CDs (1000 μg) at the nanofiber, an inhibition halo of 6.29 mm was observed (Figure 9c), slightly smaller than the 7.35 mm halo produced by gentamicin (10 μg) (Figure 9b). As illustrated in Figure 9f, the inhibition halo increased over 3 days to 7.63 mm, and to 8.54 mm over 4 days, exceeding the one-day inhibition observed for gentamicin. These results highlight the prolonged antibacterial activity of the CDs within the nanofibers, which is particularly relevant for antibacterial dressing applications, as localized antimicrobial effects may help reduce the risk of developing resistance (Figure 10).^57,58^

**Figure 9:**
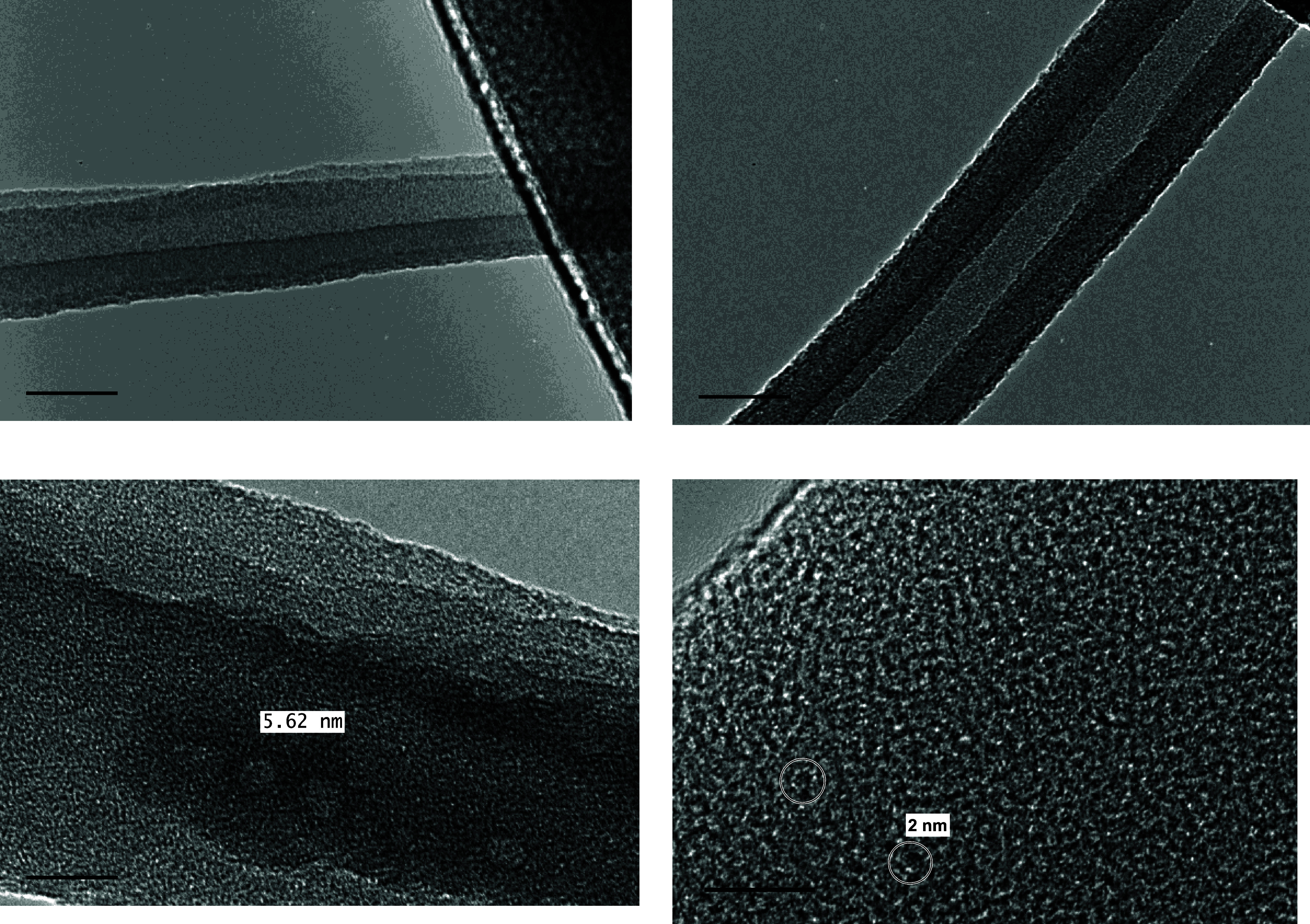
FETEM images of PVA/QS/CDs NF at different magnifications: 50 nm (top), and 20 and 10 nm (bottom).

**Figure 10:**
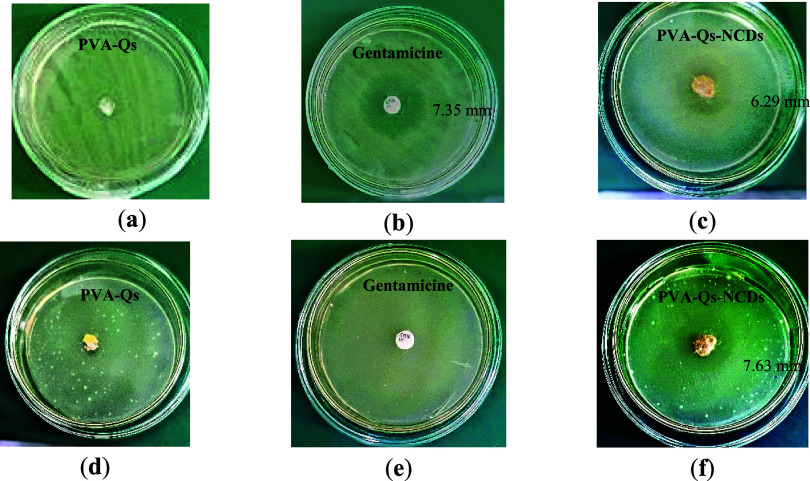
Images of the inhibition zone against *E. coli* for (a, d) PVA/QS NFs; (b, e) Gentamicin (10 μg); (c, f) PVA/QS/CDs NFs, after one and 3 days of incubation, respectively.

The antibacterial efficacy of CDs depends on their synthesis method and their surface chemistry.^59^ Various CDs have been tested against different bacterial strains, revealing significant differences. For example, CDs derived from *Taxus baccata* produced an inhibition zone (ZOI) of up to 33 mm, attributed to their high colloidal stability and efficient diffusion on agar.^60^ In contrast, CDs synthesized from Prosopis juliflora leaves produced smaller zones (∼16.7 mm), despite their positive charge, which favors electrostatic interactions with the bacterial surface. These differences illustrate that the antibacterial performance of CDs depends mainly on their physicochemical characteristics.^61^

When CDs are incorporated into polymer matrices, their diffusion is partially restricted, leading to predominantly bacteriostatic behavior. Studies of CDs in various polymer films and nanofibers demonstrate this trend.^20,62^ Table 3 summarizes representative antibacterial systems that incorporate CDs and Ag nanoparticles into polymer matrices. The data reveal several trends regarding nanoparticle (NP) type, concentration, polymer mobility, and the antibacterial evaluation method.

**Table 3:** Comparative Studies of Antibacterial Systems

polymer matrix	NPs agent	antibacterial test	*E. coli* inhibition	references
PVA film	CDs (0.5%)	agar disk diffusion	ZOI: 9.05 mm	^63^
chitosan/starch film	CDs (3%)	photodynamic inactivation (survival assay)	∼6 log reduction (after 48 h)	^64^
cellulose film	CDs (3%)	the total viable count (TVC) method	∼4.5 log reduction (after 12 h)	^65^
PAN NF	CDs	photodynamic inactivation (survival assay)	∼6 log reduction	^20^
PVA NF	Ag (10%)	agar disk diffusion	ZOI: 12 mm	^67^
PAN NF	Ag (0.05%)	agar disk diffusion	ZOI: 2.5 mm	^68^
FK/PVP/PEO NF	Ag (1.2%)	agar disk diffusion	ZOI: 8.24 mm	^69^
PVA/QS	CDs (2.5%)	agar disk diffusion	ZOI: 6.3–8.5 mm (CDs release of 1–4 days)	this work

For polymer films, free or lightly immobilized CDs generally exhibit effective antibacterial activity, although the magnitude of this effect depends strongly on CD loading. For example, PVA films containing 0.5% CDs produced an inhibition zone of 9.05 mm in the disk-diffusion assay.^63^ In comparison, QS/starch films with a higher CD content (3%) achieved ∼6-log reduction after 48 h under photodynamic activation, indicating that ROS-mediated mechanisms can significantly enhance bacterial inactivation under illumination.^64^ Cellulose films incorporating 3% CDs also exhibited potent antibacterial activity (∼4.5-log reduction at 12 h), demonstrating that hydrophilic biopolymers can facilitate CD release and interaction with bacteria.^65^ Overall, films tend to exhibit bacteriostatic or moderate bactericidal effects, determined mainly by the degree of CDs mobility within the polymer matrix and whether photoactivation is involved.

Fewer studies have examined CD-loaded nanofibers. A significant report comparing PAN nanofibers with our system, PVA/QS NF, shows a distinct antibacterial profile.^20^ While the PAN/CDs NF reported a ∼6-log reduction in bacteria during photodynamic inactivation, our system demonstrates measurable inhibition zones (6.3–8.5 mm) under ambient light conditions from days 1 to 4. Although the two metrics are not directly comparable, the results indicate that our nanofibers maintain sustained antibacterial activity without external irradiation or chemically induced cross-linking.^66^ This feature is particularly beneficial for wound-dressing applications, where extended, mild antibacterial activity is preferred over rapid, light-dependent bactericidal activity.

In contrast, nanofibers containing silver (Ag) NPs demonstrate antibacterial activity that is highly dependent on the quantity of Ag that is incorporated. For example, PVA NF with 10% Ag exhibited a zone of inhibition (ZOI) measuring 12 mm,^67^ whereas polyacrylonitrile (PAN) nanofibers with only 0.05% Ag produced a significantly smaller zone of 2.5 mm.^68^ The Keratin­(FK)/PVP/PEO NF containing 1.2% Ag attained an intermediate ZOI of 8.24 mm.^69^ These findings highlight the concentration-dependent bactericidal mechanism of silver, which is driven by the release of the Ag^+^ ions. Although silver-based systems generally outperform carbon dots (CDs) in short-term antimicrobial efficacy, concerns about cytotoxicity, environmental persistence, and regulatory considerations limit their application in the biomedical field.

In this study, agave-derived carbon quantum dots demonstrated notable antibacterial activity against *E. coli* (a Gram-negative bacterium) under ambient light conditions.^6^ While the antibacterial effect of carbon quantum dots is typically attributed to the generation of light-induced reactive oxygen species (ROS) under specific excitation wavelengths,^12^ our results suggest that additional mechanisms, such as direct interactions with bacterial membranes and contributions from surface functional groups, may also account for the observed inhibition under these conditions. However, further studies of our system for biomedical applications, such as diffusion kinetics, cytocompatibility, and long-term stability, will help to optimize its performance.

However, the biocompatibility of CDs has been documented; notably, those synthesized from natural carbon precursors tend to demonstrate low cytotoxicity and high biocompatibility due to their intrinsic surface chemistry.^70–72^ In our particular case, the CDs derived from agave carbonization exhibit abundant hydrophilic and biologically relevant functional groups (−OH, −COOH, and −NH), as confirmed by FTIR analysis. These surface characteristics are commonly associated with reduced toxicity and enhanced stability within biological environments. Additionally, the CDs display a moderately negative potential (−13 mV), which generally promotes colloidal stability and minimizes nonspecific interactions. When incorporated into the PVA/QS polymer matrix, in which the CDs are embedded within the nanofibers, the overall potential shifts to +35 mV. Nonetheless, given that the CDs are immobilized within the polymer network, their direct interaction with the biological milieu is further limited. Consequently, their release is more controlled over time, as made evident by the antibacterial assay.

## 4 Conclusions

In this study, carbon quantum dots (CDs) were successfully synthesized from the agave using the green carbonization route, yielding nanoscale particles with well-defined optical, structural, and surface characteristics. TEM, Raman, FTIR, fluorescence spectroscopy, and bandgap analysis confirmed their sp^2^-hybridized carbon core, oxygen- and nitrogen-containing functional groups derived naturally from the precursor, and blue-emitting photoluminescence associated with quantum confinement. DLS measurements revealed a hydrodynamic diameter of 4.4 nm and moderately negative ZP (−13 mV), consistent with the presence of deprotonated oxygen functionalities.

The CDs were incorporated into biodegradable PVA/QS NFs through electrospinning. The pronounced shift in ζ-potential, from negative for free CDs to strongly positive for the hybrid suspension, demonstrated electrostatic adsorption of protonated chitosan onto the CD surface under acidic conditions. This interaction not only stabilized the dispersion but also likely influenced the electrohydrodynamic behavior within the spinning jet. TEM micrographs of the nanofibers showed a darker contrast at the periphery than at the core, suggesting a preferential migration of positively charged, chitosan-coated CDs toward the fiber surface during jet elongation, resulting in a shell-like distribution.

The physicochemical characteristics of agave-derived CDs, small particle size, hydrophilic functional groups, moderate surface charge, and natural precursors have been associated with low cytotoxicity in the literature. Their incorporation into PVA/QS further limits direct biological exposure, thereby enabling a controlled, gradual release profile consistent with the wound-dressing requirements.

Overall, this work demonstrates that agave-derived CDs can be produced through a simple, environmentally friendly process and can be effectively incorporated into biodegradable nanofibrous scaffolds to achieve sustained antibacterial activity. These findings highlight their potential as sustainable and biocompatible alternatives for next-generation wound dressings. Further studies of diffusion kinetics, cytocompatibility, and long-term stability are needed to support future clinical translation.
